# Herbal medicine used for the treatment of diarrhea and cough in Kampala city, Uganda

**DOI:** 10.1186/s41182-021-00389-x

**Published:** 2022-01-07

**Authors:** Abdul Walusansa, Savina Asiimwe, Jamilu. E. Ssenku, Godwin Anywar, Milbert Namara, Jesca L. Nakavuma, Esezah K. Kakudidi

**Affiliations:** 1 Department of Plant Sciences, Microbiology and Biotechnology, School of Biosciences, Makerere University, Kampala, Uganda; 2 Department of Medical Microbiology and Immunology, Faculty of Health Sciences, Habib Medical School, Islamic University in Uganda, Kampala, Uganda; 3 Department of Biomolecular and Biolaboratory Sciences, College of Veterinary Medicine, Animal Resources and Biosecurity, Makerere University, Kampala, Uganda; 4 Department of Medical Microbiology and Immunology, Faculty of Health Sciences, Busitema University, Mbale, Uganda; 5 College of Natural and Mathematical Sciences, University of Maryland, Baltimore County, 1000 Hilltop Cir, Baltimore, MD 21250 USA

**Keywords:** Herbal medicine, Trade, Cough, Diarrhea, Herbalists, Kampala, Uganda

## Abstract

**Background:**

Globally, diarrheal and respiratory diseases are among the main causes of mortality and morbidity. In Uganda, cities are facing proliferation of trade in herbal medicines (HM), including those for diarrhea and/or cough. Information on the economic, and the ethnopharmacological aspects of these HM is scarce, deterring the sector from achieving optimal capacity to support national development. We profiled the anti-diarrhea and/or anti-cough HM, and the basic economic aspects of HM trade in Kampala city, to support ethnopharmacological knowledge conservation and strategic planning.

**Methods:**

A cross-sectional survey was conducted on 65 herbalists using semi-structured questionnaires. This was supplemented by an observational survey using a high-resolution digital camera. Data were collected following the guidelines for research on HM, established by Uganda National Drug Authority, and World Health organization.

**Results:**

Eighty-four plant species from 41 families were documented. Fabaceae and Myricaceae had the highest number of species (9, 10.7% each). *Citrus limon* (L.) Osbeck was the most commonly cited for cough, with a relative frequency of citation (RFC) of 1.00, and its relative medical importance was not significantly different from the other top 5 species except for *Azadirachta indica* A.Juss (RFC = 0.87). *Entada abyssinica* A. Rich (RFC = 0.97) was the most cited for diarrhea. Trees (34, 40.5%) were mostly used, and mainly harvested from wild habitats (55.2%) in 20 districts across Uganda. These HM were mainly sold as powders and concoctions, in markets, shops, pharmacies, and roadside or mobile stalls. The highest prices were Uganda Shillings (UGX) 48,000 ($13.15)/kg for *Allium sativum* L, and UGX 16,000 ($4.38)/kg for *C. limon*. All participants used HM trade as a sole source of basic needs; majority (60.0%) earned net monthly profit of UGX. 730,000 ($200) ≤ 1,460,000 ($400). The main hindrances to HM trade were the; disruptions caused by the COVID-19 pandemic (*n* = 65, 100%), and the scarcity of medicinal plants (58, 89.2%).

**Conclusion:**

There is a rich diversity of medicinal plant species traded in Kampala to treat diarrhea and cough. The HM trade significantly contributes to the livelihoods of the traders in Kampala, as well as the different actors along the HM value chain throughout the country.

## Background

Diarrheal and respiratory infections are among the major causes of global mortality and morbidity, triggering approximately 1.8 and 2.4 million annual deaths, respectively [1–4], especially in low and middle-income countries (LMIC) [3]. For example, in Tanzania and Uganda, diarrheal and respiratory illnesses are ranked among the six major causes of both adult and childhood mortality [4, 5]. Consequently, diarrhea and cough are the commonest syndromes for which humans seek medical care in both the rich and resource-poor countries [4, 6]. In Kampala, the capital city of Uganda, diarrhea and respiratory ailments are diseases of major concern, with estimated prevalence of 19% and 31%, respectively [7]. The infections associated with diarrhea and cough are mostly caused by microbial pathogens such as bacteria, viruses, parasites, and fungi [8–12].

The rising burden of antimicrobial resistance (AMR) is increasingly counteracting the potential of conventional medicines to manage these complications [13]. The AMR burden, coupled with other factors such as the high cost and limited availability of synthetic medicine, especially in resource-poor countries, lure most communities to resort to herbal medicine (HM) as an alternative treatment strategy [14–16]. The use of HM for healthcare needs in Uganda is estimated at 90% in rural settings [17]. In the recent decades, the trade of HM by herbalists in Uganda’s urban settings is on the rise. Herbalists are persons that have empirical medicinal plant knowledge; they offer consultation services, and/or sell HM for managing common community ailments [18]. Kampala district, being Uganda’s capital and commercial city, has a high number of herbalists compared to other urban districts [18]. This could partly be attributed to the high demand, and the lucrative market offered by the large population of residents, travelers, and the business community in the city [19]. The resident population in Kampala city mostly comprises low-income earners that live in the suburbs, and have high inclination to HM [18, 20–22].

Besides healthcare provision, the HM industry in Kampala and Uganda at large, has become one of the significant sources of employment for communities [23]. The sector provides an avenue for traditional experts to enter the urban cash economy [23, 24]. The need for strategic plans is paramount to nurture the HM trade industry to achieve its optimal capacity, and to effectively support Uganda’s fight against escalation of poverty and unemployment [25, 26]. According to the World Bank, over 21.7% of this country’s population currently live below the poverty line of US$ 1.90 per person per day [27, 28]. Also, the government of Uganda has estimated that an additional 2.6 million people could slide into extreme poverty due to the socio-economic impacts of the COVID-19 pandemic [29]. Development of the HM sector might broaden income generation for not only the herbalists, but also the other various stakeholders (e.g., farmers, collectors, transporters, and pharmacies) along the HM value chain in Uganda.

Though Kampala city is now perceived to be potentially rich in medicinal plant biodiversity stocks, the ethnopharmacological research related to these plant species is scarce. Further, the commercial aspects of HM commonly traded in Kampala city are poorly understood due to limited research on the sector. The development of urban HM trade requires comprehensive research on various aspects of the sector, such as the ethnopharmacology, and the economic aspects. The aim of this study was to document the plant species sold for the treatment of diarrhea and cough in Kampala, and information on their usage, trade, the sector-challenges. The findings could support conservation of ethnopharmacological knowledge and guide strategic planning and designing of regulatory frameworks; to enhance the potential use of HM in counteracting health burdens and poverty.

## Methods

### Study area

**Fig. 1:**
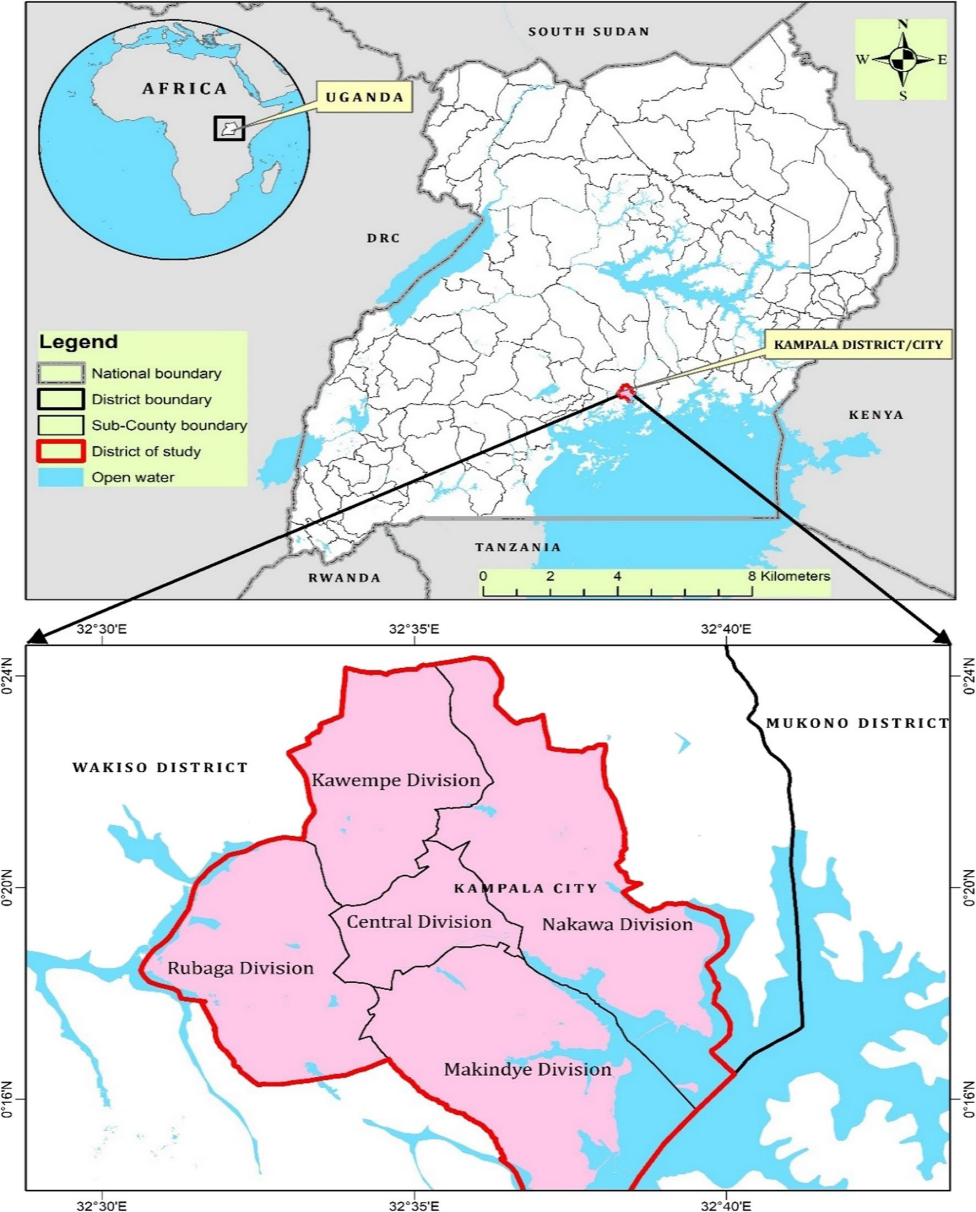
Study locale: Kampala city showing the five administrative divisions

The study was conducted in the five administrative divisions of Kampala, located in the central region of Uganda, stretching over an area between DMS Lat: 0° 12′ 46.755″ N, Long: 32° 30′ 32.567″ E and DMS Lat 0° 12′ 20.692″ N, Long: 32° 40′ 14.054″ E. It is bordered by Mukono district to the East; Lake Victoria to the South East; and Wakiso district to the West, South, and North (Fig. 1). Its five administrative divisions are; Kampala central, Kawempe, Nakawa, Makindye and Rubaga divisions. According to the recent Uganda national population census, Kampala city is populated by between 1,680,601 and 2,915,200 residential occupants [30]. In addition, a large number of individuals enter and leave the city on a daily basis either for work or as travelers [19].

### Study design and sampling technique

A cross-sectional survey was conducted on 65 traditional herbalists, between May and August 2021. Pre-tested, semi-structured questionnaires were administered to the respondents by research assistants, to collect information such as the socio-demographic profile of respondents, local names of plant species used in treatment of diarrhea and/or cough, and how they were prepared and packaged, prices, and the challenges impeding HM trade. In addition, qualitative data, such as, HM packaging patterns, categories of traditional HM outlets, and the pharmaceutical forms of HM sold, were examined through field observations which were supplemented using photography.

### Study population

The study focused on herbalists in Kampala city. The sampling frame exclusively involved herbalists that were engaged in trade, harvesting, and/or preparation of the HM.

### Sampling

#### Sample size determination

The sample size for this study was calculated by using the formula for unknown population, by Kothari [31]: *n* = *Z*^2^SD^2^/*e*^2^, where *Z* = standard error from the mean, ≈ 1.96 at 95% confidence interval; standard deviation (SD) ≈ 0.205 or 20.5% [32]; and *e* = tolerable sampling error/precision, ≈ 0.05 at 95% confidence interval. Then, the sample size was calculated as:

$$n = \frac{{\left[ {\left( {1.96} \right)^{2} \times \left( {0.205} \right)^{2} } \right]}}{{\left( {0.05} \right)^{2} }} \approx 65.$$

Therefore, 65 herbalists were recruited into this study.

#### Selection of respondents

Prior informed consent was obtained from all the participants, and ethical approval for the study was also obtained from the School of Health Sciences Research and Ethics Committee of Makerere University. Basing on the required sample size of 65 participants, 13 respondents from each of the five divisions of Kampala were recruited using a systematic random sampling approach [33]. In brief, at each of the sampling sites, the traditional healthcare units such as herbal shops and herbal-market stalls were visited, and the available population of eligible herbalists was determined through direct counting. The resultant population size was divided by the required number of respondents to deduce the sampling interval (*К*). Then, every *К*th member of the population at the respective sampling site was recruited until the required sample size was attained.

### Data collection

Pre-tested, researcher administered questionnaires were used to collect data regarding the HM used for treating diarrhea and cough in the study community. The observational survey was conducted using an observation guide, and a high-resolution digital camera inbuilt in a Phantom-9 Mobile Phone, model AB7/2019, Techno Mobile Limited [34]. All data were collected following the guidelines for research on HM products, established by the Uganda National Drug Authority (NDA), and the World Health organization [35, 36]**.**

#### Collection and identification of plant specimens

Voucher specimens of the medicinal plant species of interest were procured from HM markets and surrounding environs, in randomly selected market stalls, herbal shops, roadside stalls, and mobile stalls. The voucher specimens were pressed and transported to Makerere University Herbarium for identification. The identified plant species were authenticated according to the database at https://www.theplantlist.org, accessed on 21st Aug 2021. The plant families were checked against the Angiosperm Phylogeny Group IV.

#### Demand, supply and challenges of HM trade in Kampala city

Pre-tested, researcher administered questionnaires were used to collect data regarding the demand, supply and challenges of HM trade. This was supplemented by the observational survey which was conducted using an observation guide, and a high-resolution digital camera inbuilt in a Phantom-9 Mobile Phone, model AB7/2019, Techno Mobile Limited [34]. All data were collected following the guidelines for research on HM products, established by the Uganda National Drug Authority (NDA) and the World Health organization [35, 36].

### Data analysis

Descriptive, and inferential statistics like frequencies, percentages, and Chi-square were used to analyze the data. The Relative Frequency of Citation (RFC) was used to evaluate the ethnopharmacological data. The data were analyzed using STATA version-15.0 software.

#### Relative frequency of citation (RFC)

The relative frequency of citation (RFC) for each HM was computed to determine the number of herbalists that considered particular plant species as being worth mentioning in the management of diarrhea and cough. The RFC values range between 0 and 1 (where 1 indicates the highest level of respondents’ consensus on the use of that species to manage a particular disease). The value was calculated using a formular described by Tardio and Santayana [37]:

$${\text{RFC}} = \frac{{{\text{FCs}}}}{N} = \mathop \sum \limits_{i = i1}^{iN} uRi/N,$$

where *FC* is the number of herbalists who cited a particular species, and *N* is the total number of herbalists (Table 2).

## Results

### Socio-demographic profile of participants

Majority (*n* = 36, 55.4%) of the respondents recruited were men, while females constituted 44.6% (*n* = 29). Most of the participants were in the age categories of the youths (*n* = 24, 36.9%), and the middle-aged (*n* = 39, 60%); only two (3.1%) were elderly. The majority of the participants (*n* = 26, 40.0%) had attended secondary education, while three (4.6%) had attained tertiary education. The participants who had practiced traditional medicine for a duration of between 5 and 15 years constituted 72.3% (*n* = 47). Sixty-five (100%) participants unanimously perceived the importance of medicinal plants and the need to trade these remedies, viz; all participants indicated that they generated significant profits to meet their basic livelihood needs, and that they were optimistic about the future of herbal medicine trade in Uganda. A net monthly profit of UGX 730,000 ($200) ≤ 1,460,000 ($400) was earned by 39 (60.0%), while 5 (7.7%) earned above UGX. 1,825,000 ($500) from HM sales (Table 1).

**Table 1:** Socio-demographic characteristics of commercial herbalists in Kampala city (*N* = 65)

Variable	Frequency, *n* (%)
Gender	
Male	36 (55.4)
Female	29 (44.6)
Age (years)	
18–24 (youths)	24 (36.9)
25–63 (middle-aged)	39 (60.0)
≥ 64 (elderly)	2 (3.1)
Nationality	
Ugandan	65 (100)
Non-Ugandan	0 (0.0)
Marital status	
Married	39 (60.0)
Single	26 (40.0)
Education	
None	8 (12.3)
Primary	26 (40.0)
Secondary	28 (43.1)
Tertiary	3 (4.6)
Years of experience in HM	
5 ≤ 15	47 (72.3)
16 ≤ 20	15 (23.1)
> 20	3 (4.6)
Type of HM establishment	
Roadside stalls	18 (27.7)
Market stalls	18 (27.7)
Herbal shops	17 (26.2)
Mobile stalls	12 (18.4)
Estimated monthly net profit from HM, UGX (USD)	
< 730,000 (200)	9 (13.8)
730,000 ≤ 1,460,000 (400)	39 (60.0)
1,460,000 < 1,825,000 (500)	12 (18.5)
≥ 1,825,000 (500)	5 (7.7)

*UGX* Uganda shillings, *$* United States dollar, *HM* herbal medicine

### Diversity of medicinal plants traded for treatment of diarrhea and cough in Kampala

A total of 84 medicinal plant species belonging to 41 families and 73 genera used in the management of diarrhea and cough were documented in the commercial HM establishments surveyed (Table 2). The families; Fabaceae and Myricaceae were the most dominant; contributing 9, (10.7%) species each. These were followed by family Asteraceae (7, 8.3%). Forty-four species (52.4%) were cited for diarrhea treatment, 31, (36.9%) for cough, and 9, (10.7%) for both ailments. Most of the plant species cited were trees (34, 40.5%) (Table 2).

**Table 2:** Medicinal plants used for treatment of diarrhea and cough in Kampala city, Uganda

Family, voucher no.	Serial no., local name	Scientific name	Life form	Parts used	Mode of administration	RFC
Species used against cough						
Alliaceae						
KHM03	1. Katungulchumu^a^ Tungulucumu^d^	*Allium sativum* L.	Herb	Bulb	Decoction + minced ginger, drunk	0.64
Asparagaceae						
KHM05	2. Kajjolyenjovu^a^	*Dracaena steudneri* Engl.	Tree	Leaves	Decoction drunk/powder licked	0.07
Astareceae						
KHM06	3. Artemesia^k^	*Artemisia annua* L.	Herb	Leaves	Infusion + salt drunk	0.03
KHM07	4. Mululuuza^a^	*Vernonia amygdalina* Delile	Shrub	Leaves, roots	Decoction drunk	0.61
Bignoniaceae						
KHM36	5. Mussa^ab^	*Kigelia africana* (Lam.) Benth.	Tree	Leaves	Decoction + honey drunk	0.09
KHM37	6. Kifabakazi^a^	*Spathodea campanulata* P.Beauv.	Tree	RB	Decoction drunk	0.04
Caricaceae						
KHM08	7. Mupapaali^b^	*Carica papaya* L.	Tree	Leave, roots	Decoction drunk	0.10
Celastraceae						
KHM09	8. Mayirunji^a^	*Catha edulis* Forssk.	Shrub	Leaves	Chewed, extract swallowed	0.02
KHM10	9. Muwaiswa^c^	*Gymnosporia senegalensis* (Lam.) Loes.	Shrub	Roots, leaves	Decoction drunk	0.02
KHM14	10. Musaali^a^	*Symphonia globulifera* L.f.	Tree	Roots	Decoction drunk	0.03
Crassulaceae						
KHM15	11. Kiyondo Ekyeru^a^	*Kalanchoe densiflora* Rolfe	Herb	Leaves	Decoction drunk	0.06
Cucurbitaceae						
KHM11	12. Suunsa^a^	*Cucurbita maxima* Duch.	Creeper	Leaves	Decoction drunk	0.11
Ebenaceae						
KHM16	13. Mangholu^e^	*Euclea schimperi* (A.DC.) Dandy	Shrub	Leaves	Decoction drunk twice a day before meals	0.02
Fabaceae						
KHM17	14. Akasaana^a^	*Acacia hockii* De Wild.	Shrub	SB	Decoction drunk	0.05
KHM20	15. Nkooge^a^	*Tamarindus indica* L.	Tree	Fruit, SB, leaves	Decoction drunk	0.18
Dracaenaceae						
KHM19	16. Akasandasanda^a^	*Euphorbia hirta* Lnn.	Herb	Leaves	Decoction drunk	0.06
Rubiaceae						
KHM18	17. Odwong^h^	*Gardenia ternifolia* Schumach. & Thonn. subsp. jovis-tonantis (Welw.) Verdc. var. jovis-tonantis	Tree	Root bark	Infusion of dry powder drunk	0.02
Lamiaceae						
KHM01	18. Kyewamala^a^	*Tetradenia riparia* (Hochst.) Codd	Shrub	Leaves	Infusion drunk	0.22
KHM02	19. Kibwankulata^a^	*Plectranthus cyaneus* Gürke	Herb	Leaves	Decoction drunk	0.39
KHM21	20. Kachumita^d^	*Basilicum polystachyon* (L.) Moench	Herb	Leaves	Decoction drunk	0.02
Lauraceae						
KHM22	21. Ovakedo^a^	*Persea americana* Mill.	Tree	Leaves, SD, SB	Decoction drunk	0.66
Malvaceae						
KHM23	22. Lusaala^a^	*Hibiscus fuscus* Garcke	Herb	Leaves	Ash licked	0.08
Moraceae						
KHM24	23. Muvule^a^	*Miliciaxcels* (Welw.) C.C.Berg	Tree	Leaves, SB	Decoction drunk	0.21
Moringaceae						
KHM25	24. Molinga^a^	*Moringa oleifera* Lam.	Tree	Leaves, roots, SD	Decoction drunk	0.32
Myricaceae						
KHM26	25. Nkikimbo^a^	*Morella kandtiana* (Engl.) Verdc. & Polhill	Shrub	Roots	Decoction drunk	0.02
KHM13	26. Kalitunsi^a^	*Eucalyptus grandis* W. Hill	Tree	Leaves, SB	Infusion drunk	0.74
KHM62	27. Kalatuc^i^	*Eucalyptus viminalis* Labill.	Tree	Leaves, RB	Decoction drunk	0.06
KHM27	28. Mwambalabutonya^a^	*Callistemon citrinus* (Curtis) Skeels	Shrub	Leaves	Decoction drunk	0.95
KHM28	29. Kalitunsi^a^	*Corymbia citriodora* (Hook.) K.D.Hill & L.A.S.Johnson	Tree	Leaves, SB	Concoction drunk	0.05
KHM29	30. Jjambula^ac^	*Syzygium cumini* (L.) Skeels	Tree	Leaves	Decoction drunk	0.66
KHM30	31. Mupeera^a^	*Psidium guajava* L.	Tree	Leaves	Decoction drunk	0.75
Rutaceae						
KHM04	32. Niimu^a^ Ndima^f^	*Citrus limon* (L.) Osbeck	Shrub	Fruits	Decoction of whole fruit/infusion of fresh mesocarp drunk, or juice squeezed out and swallowed	1.00
Species used against diarrhea						
Acanthaceae						
KHM41	33. Wankuura^d^	*Thunbergia alata* Bojer ex Sims	Climber	Leaves	Decoction drunk	0.02
Anacardiaceae						
KHM43	34. Muziru^a^	*Pseudospondias microcarpa* (A. Rich) Engl.	Tree	Roots	Decoction drunk	0.22
Annonaceae						
KHM44	35. Mugaali^a^	*Annona senegalensis* Pers.	Tree	SB, leaves	Decoction drunk	0.02
Apocynaceae						
KHM45	36. Mulondo^ab^	*Mondia whytei* (Hook.f.) Skeels	Climber	Roots	Infusion/chew	0.49
Aristolochiaceae						
KHM39	37. Nakasero^a^ Musujja awalaba^d^	*Aristolochia littoralis* Parodi	Herb	Leaves	Decoction drunk	0.70
Astareceae						
KHM46	38. Akalulusa ahasinde^e^	*Microglossa angolensis* Oliv. & Hiern.	Shrub	Leaves	Decoction drunk	0.03
KHM47	39. Kafugankande^a^	*Conyza pyrrhopappa* Sch.Bip. ex A. Rich	Herb	Leaves	Decoction drunk	0.95
KHM12	40. Etutum^h^	*Microglossa pyrifolia* (Lam.) O.Kuntze	Herb	Roots, leaves	Decoction drunk	0.09
KHM48	41. Mugango^a^	*Solanecio mannii* (Hook.f.) C.Jeffrey	Herb	Leaves	Decoction drunk	0.04
KHM42	42. Ssere^a^	*Bidens pilosa* L.	Herb	Leaves	Decoction drunk	0.33
Balanitaceae						
KHM49	43. Liggwa limu^a^	*Balanites aegyptiaca* (L) Delile	Tree	Roots	Decoction drunk	0.11
Burseraceae						
KHM50	44. Muwafu^a^	*Canarium schweinfurtii* Engl.	Tree	SB	Decoction drunk	0.26
Capparaceae						
KHM51	45. Mukolokombi^a^	*Capparis tomentosa* Lam.	Shrub	Roots	Decoction drunk	0.04
Convolvulaceae						
KHM53	46. Lumonde^a^	*Ipomoea batatas* (L.) Lam.	Vine	Leaves	Decoction drunk	0.31
Crassulaceae						
KHM52	47. Kiyondo^a^	*Bryophyllum pinnatum* (Lam.) Oken	Herb	Leaves	Decoction drunk	0.41
Euphorbiaceae						
KHM69	48. Ahadunga^e^	*Euphorbia heterochroma* Pax	Tree	SB	Decoction drunk	0.04
KHM55	49. Murangara^d^	*Croton macrostachyus* Hochst. ex Delile	Tree	Leaves	Decoction drunk	0.02
Fabaceae						
KHM56	50. Lusiiti^ab^	*Abrus precatorius* L.	Tree	Leaves	Decoction drunk	0.13
KHM57	51. Muwologoma^a^	*Acacia amythethophylla* A. Rich.	Shrub	Roots	Decoction drunk	0.17
KHM58	52. Katasubwa^b^	Acacia senegal (L.) Willd.	Shrub	Roots	Decoction	0.06
KHM59	53. Mugavu^a^ Kiluku^j^	*Albizia coriaria* Oliv	Tree	Stem bark	Decoction drunk	0.35
KHM60	54. Nkolimbo^ab^	*Cajanus cajan* (L.) Millsp.	Herb	Leaves	Decoction/infusion drunk	0.24
KHM61	55. Jjirikiti^a^	*Erythrina abyssinica* DC.	Tree	SB, roots	Decoction drunk	0.05
KHM67	56. Kiyugeyuge^b^	*Tylosema fassoglensis* (Schweinf.) Torre & Hillc.	Climber	Roots	Concoction drunk	0.02
Lauraceae						
KHM66	57. Mukomamawananga^a^	*Punica granatum* L.	Shrub	SB	Decoction drunk	0.02
Meliaceae						
KHM65	58. Musonko^a^	*Lovoa trichilioides* Harms	Tree	SB, SD, leaves	Infusion drunk	0.05
Moraceae						
KHM64	59. Mutuba^a^	*Ficus natalensis* Hochst.	Tree	SB	Decoction drunk	0.16
Myricaceae						
KHM63	60. Kalitunsi^a^	*Eucalyptus globulus* Labill.	Tree	SB, leaves	Decoction drunk	0.03
KHM68	61. Kalitunsi^a^	*Eucalyptus saligna* Sm.	Tree	Leaves	Decoction drunk	0.06
Onagraceae						
KHM70	62.Kajampuni^c^ Kanyebwa^a^	*Oxalis latifolia* Kunth	Herb	Shoot	Decoction drunk	0.24
Peraceae						
KHM72	63. Mubarama^d^	*Clutia abyssinica* Jaub. & Spach	Shrub	Leaves	Infusion/decoction drunk	0.02
Phyllanthaceae						
KHM31	64. Katazamiti^a^	*Bridelia micrantha* (Hochst.) Baill	Tree	Leaves, SB	Decoction drunk	0.06
KHM32	65. Mutulika^a^	*Phyllanthus ovalifolus* Forssk.	Shrub	Leaves	Decoction drunk	0.05
Poaceae						
KHM71	66.Ekyisubi^a^	*Cymbopogon flexuosus* (Nees ex Steud.) W. Watson	Grass	Leaves	Infusion drunk	0.96
KHM73	67. Lumbugu^a^	*Digitaria abyssinica* (A. Rich.) Stapf	Grass	Leaves	Decoction drunk	0.48
Polygalaceae						
KHM33	68. Mukondwe^a^	*Securida longipedunculata* Fresen.	Tree	Roots, leaves	Concoction drunk	0.11
Portulacaceae						
KHM74	69. Muhanga^d^	*Maesa lanceolata* Forssk.	Tree	SB	Decoction drunk	0.02
Rosaceae						
KHM34	70. Ntaseesa^b^ Ngwabuzito^a^	*Prunus africana* (Hook.f.) Kalkman	Tree	SB, leaves	Decoction drunk	0.13
KHM38	71. Ensaali^a^	*Eriobotrya japonica* (Thumb) Lindl.	Shrub	Leaves	Decoction drunk	0.08
Lamiaceae						
KHM75	72. Mujaaja^a^	*Ocimum gratissimum* L.	Herb	Leaves	Decoction drunk	0.04
KHM40	73. Mubolo^a^	*Citropsis articulata* (Willd. ex Spreng.) Swingle & M.Kellerm	Shrub	SB	Decoction drunk	0.07
Verbenaceae						
KHM76	74. Enkami^a^	*Priva flabelliformis* (Moldenke) R. Fern.	Herb	Leaves	Decoction drunk	0.47
Zingiberaceae						
KHM35	75. Ntangawuzi^a^	*Zingiber officinale* Roscoe	Herb	Rhizome	Tincture drunk	0.14
Species used against both diarrhea and cough						
Anacardiaceae						
KHM54	76. Muyembe^a^ Mengu^f^	*Mangifera indica* L.	Tree	Leaves	Decoction drunk	0.75^C^, 0.21^D^
KHM77	77. Kakwansokwanso^a^	*Searsia pyroides* (Burch.) Moffett	Shrub	Leaves, Roots	Decoction drunk	0.19^C^, 0.07^D^
Canellaceae						
KHM79	78. Omuya^a^	*Warburgia ugandensis* Sprague	Tree	Leaves, SB, roots	Decoction/infusion drunk	0.03^D^, 0.08^C^
Cucurbitaceae						
KHM83	79. Bombo^a^ Bomo^g^	*Momordica foetida* Schumach	Climber	Leaves	Infusion drunk	0.98^C^, 0.40^D^
Meliaceae						
KHM81	80. Neem^k^	*Azadirachta indica* A.Juss.	Tree	Roots, leaves, SB	Decoction drunk	0.87^C^, 0.03^D^
Passifloraceae						
KHM82	81. Katunda^ad^	*Passiflora edulis* Sims	Climber	Leaves	Decoction drunk	0.04^D^, 0.26^C^
Poaceae						
KHM80	82. Teete^a^	*Cymbopogon citratus* Stapf	Grass	Leaves	Decoction or infusion drunk	0.32^C^, 0.06^D^
Rutaceae						
KHM78	83. Muchungwa^ab^ Chungwa^g^	*Citrus sinensis* (L.) Osbeck	Shrub	Leaves, roots, SB, fruit	Decoction drunk	0.03^D^, 0.71^C^
Fabaceae						
KHM84	84. Mwolola^a^	*Entada abyssinica* A. Rich	Tree	SB, leaves	Infusion/decoction drunk	0.97^D^, 0.20^C^

*D* diarrhea, *C* cough, *SB* stem bark, *SD* seeds, *RB* root bark

Languages spoken: ^a^Luganda, ^b^Lusoga, ^c^Lugishu, ^d^Runyankore, ^e^Lunyole, ^f^Lugbara, ^g^Langi, ^h^Ateso, ^i^Luo, ^j^Ik/Karamojong, ^k^local name not available

### Methods of preparation and modes of administration

**Fig. 2:**
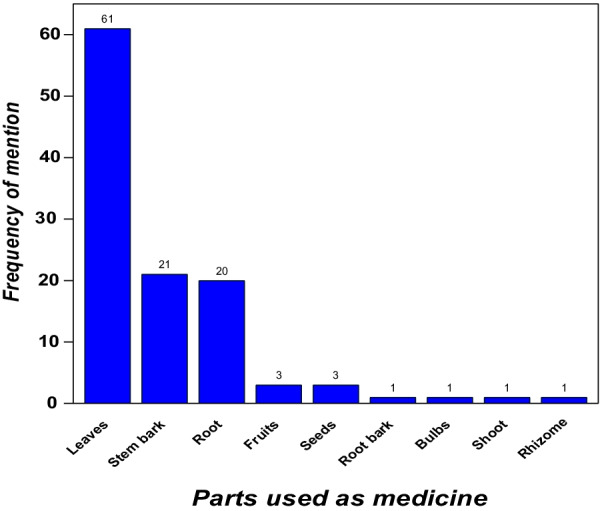
Medicinal plant parts used to treat diarrhea and cough in Kampala city

All (84, 100%), of the plant species recorded were administered orally, in four main forms, that is, decoctions (*n* = 70, 83.3%), infusions (*n* = 13, 15.5%), powders licked (*n* = 2, 2.4%), and fresh plant materials chewed (*n* = 2, 2.4%) (Table 2). Leaves were the major plant part used (*n* = 61, 93.8%) followed by the stem bark (*n* = 21, 32.3%) (Fig. 2).

### Highly traded species for management of diarrhea and cough in Kampala

**Fig. 3:**
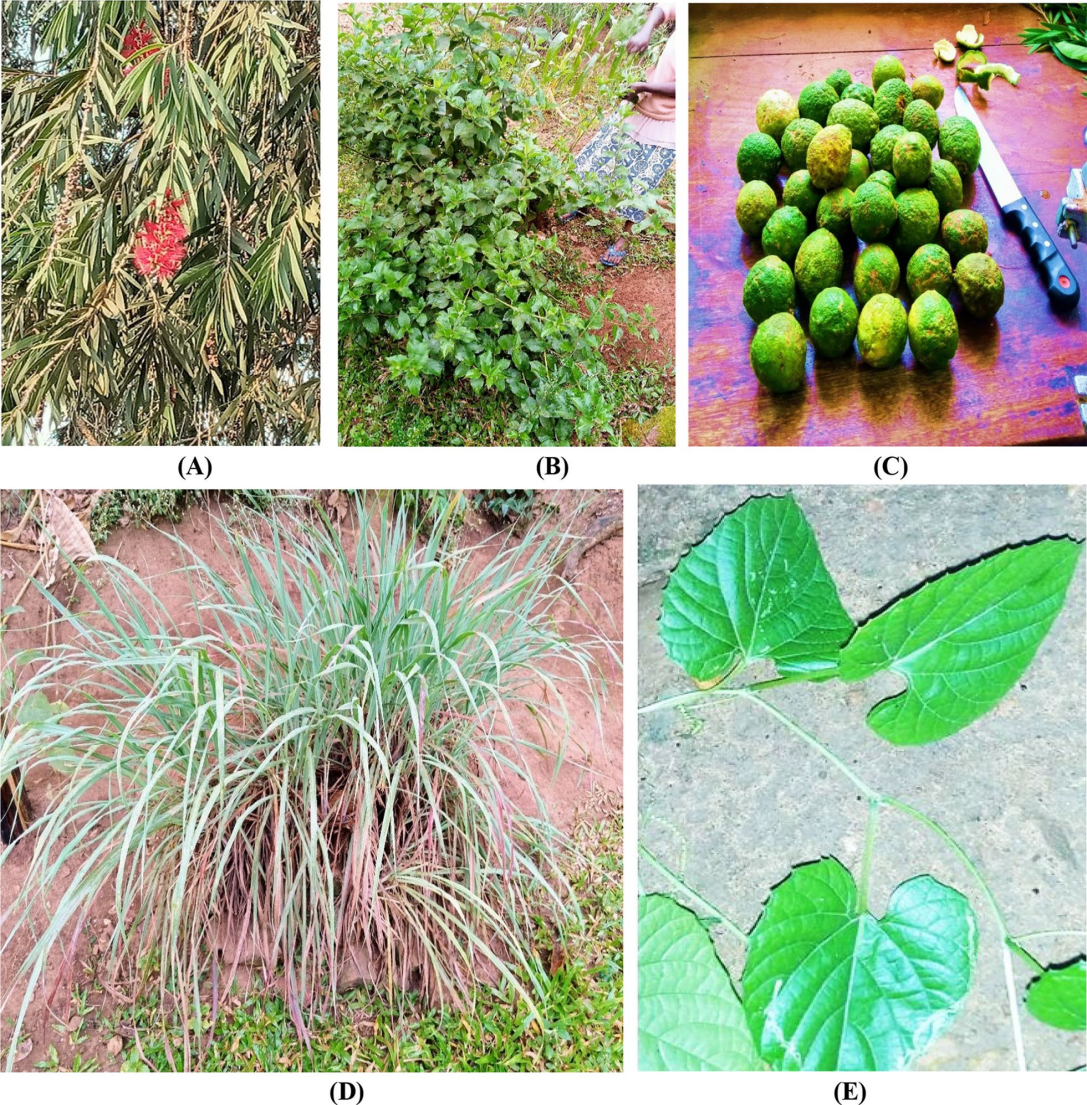
Some frequently used plant species in the management of diarrhea and/or cough in Kampala city. **A** *C. citrinus*; **B** *C. pyrrhopappa*; **C** *C. limon* fruits; **D** *C. flexuosus*; **E** *M. foetida*

*Citrus limon* attained the highest RFC of 1.00 for cough treatment, followed by *M. foetida* (RFC = 0.98). *E. abyssinica* was the most highly cited HM in the management of diarrhea (RFC = 0.97). *C. edulis* and *G. senegalensis* were least mentioned (each with RFC = 0.02) for cough treatment, while *T. fassoglensis*, and *P. granatum* were least mentioned for diarrhea treatment. The most frequently cited plant species (RFC ≥ 0.70) are summarized in Table 3 and some illustrated in Fig. 3a–e.

**Table 3:** Species that are frequently used for diarrhea and cough treatment in Kampala

Plant species	Disease treated	RFC	*χ*^2^	*p*-value	Previous reports on diarrhea and/or cough treatment
*Citrus limon*	Cough	1.00 REF			[95]
*Momordica foetida*	Cough	0.98	1.303	0.2537	[96]
*Entada abyssinica*	Diarrhea	0.97	1.964	0.1611	[56]
*Cymbopogon flexuosus*	Diarrhea	0.96	2.631	0.1048	[97]
*Callistemon citrinus*	Cough	0.95	3.305	0.0691	[56, 48]
*Conyza pyrrhopappa*	Diarrhea	0.95	3.305	0.0691	[98]
*Azadirachta indica*	Cough	0.87	8.923	0.0028	[99]
*Psidium guajava*	Cough	0.75	18.047	0.0001	[100]
*Mangifera indica*	Cough	0.75	18.047	0.0001	[101]
*Eucalyptus grandis*	Cough	0.74	18.830	0.0001	[49]
*Citrus sinensis*	Cough	0.71	21.232	0.0001	[49]
*Aristolochia littoralis*	Diarrhea	0.70	22.068	0.0001	[52]

*χ*^2^: Chi-square; RFC: relative frequency of citation; REF: reference value

### Herbal medicine trade in Kampala city

#### Sources of herbal medicines traded in Kampala

**Fig. 4:**
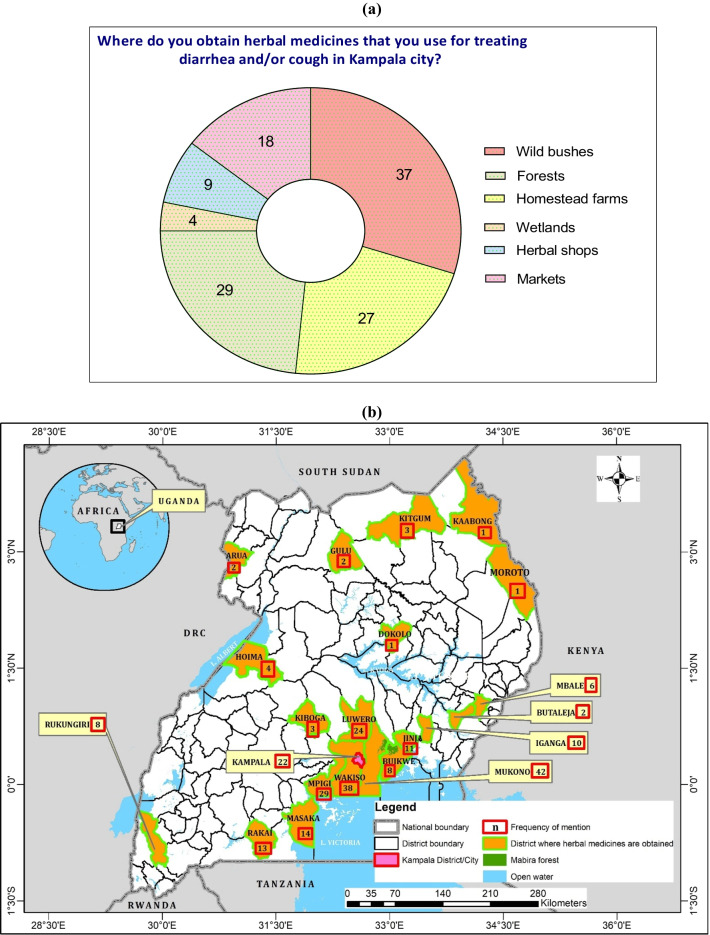
**a**, **b** Sources of HM traded by herbalists in Kampala

Among the 65 herbalists interviewed, 59 (90.8%) had information about of the habitats from where the medicinal plants were harvested. Although the rest were knowledgeable about the HM they sold, they were unable to provide information about the habitats of some plants. These participants attributed the knowledge-deficit to the fact that they often purchased most of the HM from fellow herbalists who were whole sellers, hence minimal knowledge on the natural settings from where some of the HM were sourced. Most participants claimed to obtain the HM from wild habitats such as bushes (56.9%) and forests (44.6%) (Fig. 4a). Most HM were sourced from Mukono (64.6%) and Wakiso (58.5%) districts, which boarder Kampala City (Fig. 4b). Only 16.9% (*n* = 11) of the participants identified the source of their raw materials as Mabira central Forest Reserve, which covers the districts of Mukono, Jinja and Buikwe in the Central and Eastern Uganda (Fig. 4b).

#### Types of traditional healthcare establishments where HM are traded

**Fig. 5:**
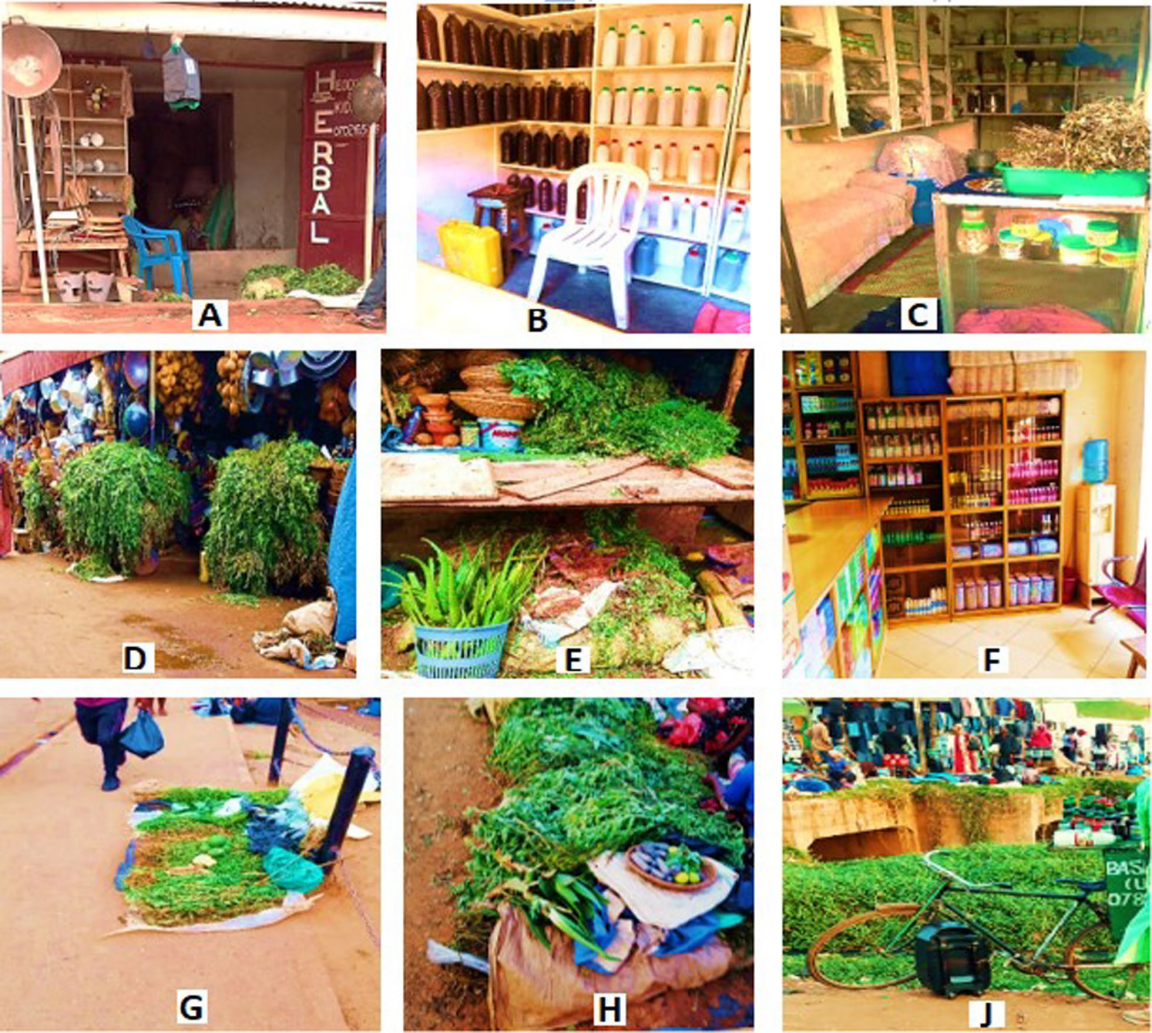
Some traditional health care establishments in Kampala city, where herbal medicines are traded: formal HMSE (**A**–**C** herbal shops; **D**, **E** market stalls; **F** pharmacy). Informa HMSE (**G**, **H** roadside stalls; **J** mobile stalls)

The herbal medicine-selling establishments (HMSE) were classified into two major categories namely; (i) formal and (ii) informal. The formal establishments included the HMSE found in places gazetted for trade by the government of Uganda. In this category, three main types were observed namely; herbal shops, market stalls, and pharmacies. The informal HMSE included roadside stalls, and mobile stalls (Fig. 5A–J).

#### Pharmaceutical forms and packaging of herbal medicines traded in Kampala city

**Fig. 6:**
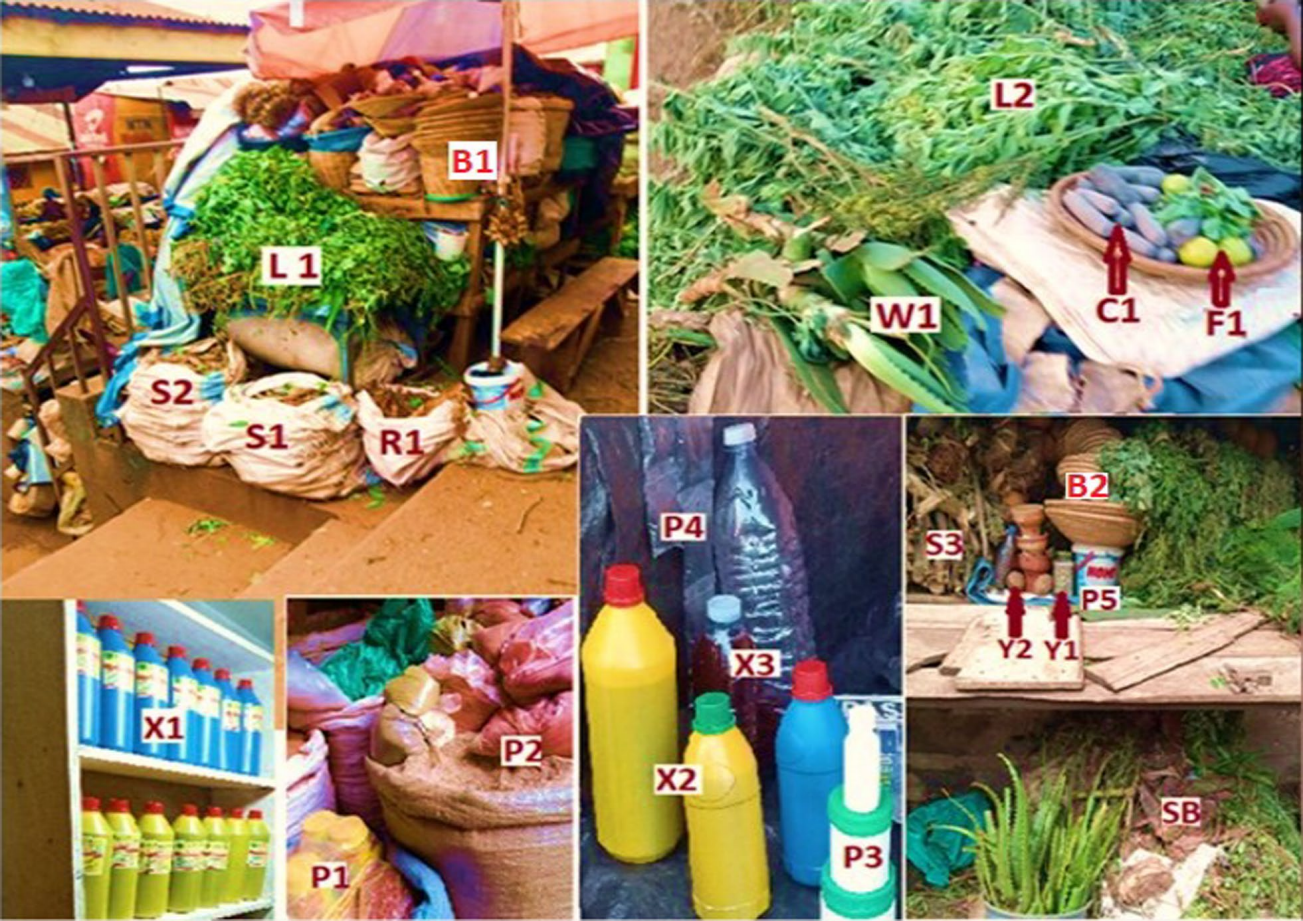
**a** Pharmaceutical forms of commercial HM in Kampala: (i) Herbal Medicine Products [Liquid preparations (X1, X2, X2), Powders (P1, P2, P4, P5), Gels (P3), Herbal extracts concocted in clay (C1)]. (ii) Herbal substances [Leaves (L1), Stems (S1, S2, S3), Roots (R1), Whole plant (W1, L2), Fruits (F1), Seeds (Y1, Y2), Stem barks (SB)]. **b** Categories of HM packaging material: (i) Original packages [Plastic bottles (X1, X2), Polyethene bags (P2, P4), Tins (P3)], (ii) Recycled packages [Sacks (S1, S2, R1), Bottles (X3), Buckets (P5), Baskets (B1, B2)]

The HM were presented in two broad categories namely: (a) herbal medicine products (defined as finished, labeled medicinal products containing active ingredients in form of plant parts. These may be in crude state, or as preparations, or in combination with other excipients which are not of plant origin); and (b) herbal substances (defined as either whole or fragments of fresh or dry plants that have not been subjected to isolation and purification of active ingredients) [35] (Fig. 6). The packaging of HM fell into three categories namely; (i) original packaging materials (bought from manufacturer/supplier and had never been used for other purposes); (ii) Recycled/re-used packages (previously used for other purposes), and (iii) non-packaged (plainly displayed for sale) (Fig. 6).

#### Demand and supply of commonly traded herbal medicine used for treating diarrhea and cough in Kampala

Among the 84 plant species identified in this survey, 15 were categorized as commonly used by virtue of having high relative frequency of citation (RFC ≥ 0.70). The rate at which these HM were purchased was also examined, and categorized as: high (H), moderate (M), and low (L). The majority (8, 53.3%) were highly demanded, while five species were on low demand (Table 4). Declining availability in the natural habitats was reported for nine (60%) of the frequently used species (Table 4). Except for *E. grandis*, all the 15 most frequently mentioned species were reported to be out of stock in ≥ 3 traditional healthcare establishments during the survey. The most expensive plant species (fresh material), were: *A. sativum* sold at UGX 48,000 ($13.15), *C. limon* at UGX 16,000 ($4.38) and *E. grandis* at UGX 13,500 ($3.70) per kilogram, respectively. The least priced were *P. americana*, and *S. cumini* each sold at UGX 4000 ($1.10) per kilogram (Table 4).

**Table 4:** Availability, demand and prices of frequently traded diarrhea and cough herbals in Kampala (*N* = 65)

Species name	Disease treated	Availability in HM selling premises during survey	Demand	Availability in natural habitats	Average price, UGX (USD)/kg^a^
*Citrus limon*	Cough	Available (*n* = 28, 43.1%) Out of stock (*n* = 37, 56.9%)	H (*n* = 65, 100%)	Declining (*n* = 16, 24.6%) Rare (*n* = 49, 75.4%)	16,000 (4.38)
*Momordica foetida*	Cough, Diarrhea	Available (*n* = 50, 76.9%) Out of stock (*n* = 9, 14.1%)	H (*n* = 59, 91%)	Rare (*n* = 59, 91.0%)	11,000 (3.01)
*Callistemon citrinus*	Cough	Available (*n* = 47, 72.3%) Out of stock (*n* = 10, 15.7%)	H (*n* = 57, 88%)	Declining (*n* = 52, 80%) Rare (*n* = 5, 0.08%)	10,000 (2.74)
*Entada abyssinica*	Cough, Diarrhea	Available (*n* = 30, 46.2%) Out of stock (*n* = 10, 15.7%)	M (*n* = 58, 89.2%) R (*n* = 5, 0.77)	Declining (*n* = 3, 4.6%) Rare (*n* = 60, 92.3%)	7000 (1.92)
*Cymbopogon flexuosus*	Diarrhea	Available (*n* = 18, 27.7%) Out of stock (*n* = 37, 56.3%)	M (*n* = 11, 16.9%) L (*n* = 44, 67.7%)	Declining (*n* = 55, 84%)	9500 (2.60)
*Conyza pyrrhopappa*	Diarrhea	Available (*n* = 7, 20.5%) Out of stock (*n* = 46, 61.5.3%)	M (*n* = 5, 0.37%) L (*n* = 48, 78.3%)	Rare (*n* = 53, 82%)	6000 (1.64)
*Azadirachta indica*	Cough	Available (*n* = 16, 24.7%) Out of stock (*n* = 34, 52.3%)	H (*n* = 50, 77%)	Declining (*n* = 50, 77%)	10,000 (2.74)
*Psidium guajava*	Cough	Available (*n* = 31, 47.7%) Out of stock (*n* = 18, 27.7%)	H (*n* = 10, 15.4%) M (*n* = 39, 59.6%)	Abundant (*n* = 42, 64.6%) Declining (*n* = 7, 10.4%)	7000 (1.92)
*Mangifera indica*	Cough	Available (*n* = 44, 67.7%) Out of stock (*n* = 4, 7.3%)	M (*n* = 48, 75.0%)	Abundant (*n* = 48, 75.0%)	5000 (1.37)
*Eucalyptus grandis*	Cough	Available (*n* = 48, 74.0%)	H (*n* = 48, 74.0%)	Declining (*n* = 48, 74.0%)	13,500 (3.70)
*Allium sativum*	Cough	Available (*n* = 29, 44.6%) Out of stock (*n* = 12, 19.4%)	H (*n* = 41, 64%)	Abundant (*n* = 7, 10.8%) Declining (*n* = 8, 12.3%) Rare (*n* = 26, 40.9%)	48,000 (13.15)
*Vernonia amygdalina*	Cough	Available (*n* = 36, 55.4%) Out of stock (*n* = 4, 5.6%)	M (*n* = 40, 61%)	Abundant (*n* = 31, 47.7%) Declining (*n* = 9, 13.3%)	5000 (1.37)
*Persea americana*	Cough	Available (*n* = 27, 41.5%) Out of stock (*n* = 16, 24.5%)	M (*n* = 6, 9.2%) L (*n* = 37, 56.8%)	Abundant (*n* = 43, 66.0%)	4000 (1.10)
*Syzygium cumini*	Cough	Available (*n* = 30, 46.2%) Out of stock (*n* = 13, 19.8%)	M (*n* = 8, 12.3%) L (*n* = 35, 53.7%)	Abundant (*n* = 43, 66.0%)	4000 (1.10)
*Citrus sinensis*	Cough	Available (*n* = 43, 66.2%) Out of stock (*n* = 3, 4.8%)	H (*n* = 46, 71.0%)	Declining (*n* = 46, 71.0%)	8000 (2.19)
*Aristolochia littoralis*	Diarrhea	Available (*n* = 20, 30.8%) Out of stock (*n* = 25, 39.2%)	M (*n* = 12, 18.5%) L (*n* = 33, 51.5%)	Abundant (*n* = 20, 30.8%) Rare (*n* = 25, 39.2%)	5500 (1.51)

*UGX* Uganda shillings, *USD* United States dollars, *kg* kilogram, *HM* herbal medicine, *H* high, *M* moderate, *L* low

^a^Average exchange rate of USD 1.0 = UGX 3650 [102]

#### Challenges associated with herbal medicine trade in Kampala city

**Fig. 7:**
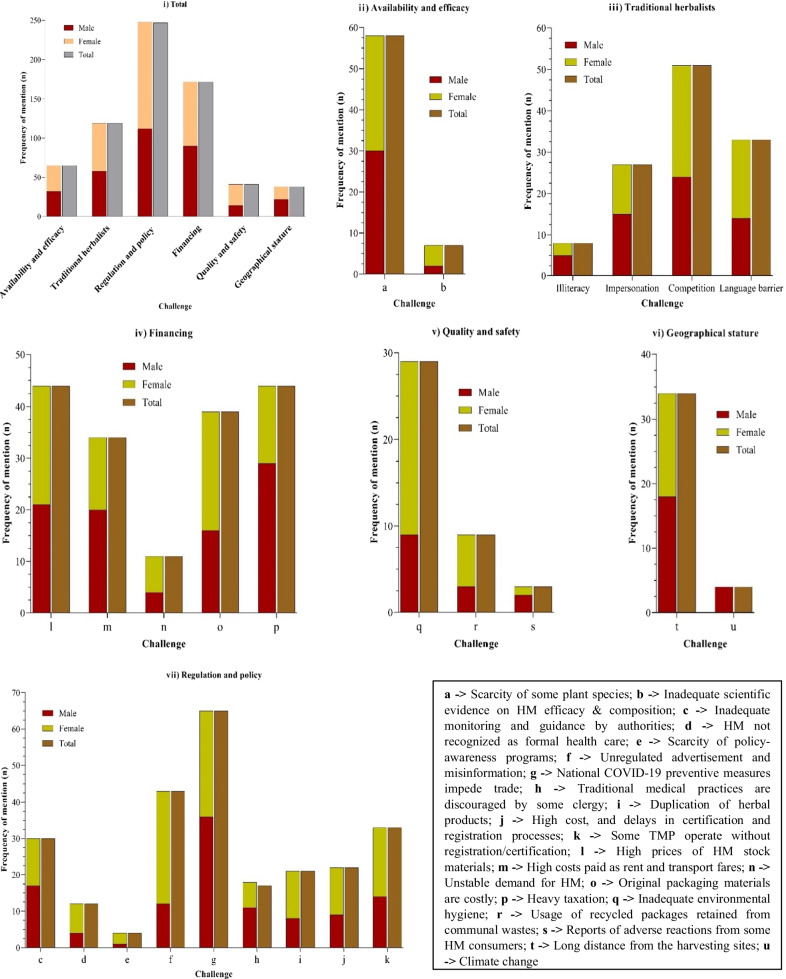
Herbal medicine trade challenges in Kampala city (*n* = 65). a Scarcity of some plant species; b Inadequate scientific evidence on HM efficacy &composition; c Inadequate monitoring and guidance by authorities; d HM not recognized as formal health care; e Scarcity of policy-awareness programs; f Unregulated advertisement and misinformation; g National COVID-19 preventive measures impede trade; h Traditional medical practices are discouraged by some clergy; i Duplication of herbal products; j High cost, and delays in certification and registration processes; k Some TMP operate without registration/certification; l High prices of HM stock materials; m High costs paid as rent and transport fares; n Unstable demand for HM; o Original packaging materials are costly; p Heavy taxation; q Inadequate environmental hygiene; r Usage of recycled packages retained from communal wastes; s Reports of adverse reactions from some HM consumers; t Long distance from the harvesting sites; u Climate change

The herbalists interviewed in this study reported 25 challenges (Fig. 7). HM trade challenges were grouped into six themes using thematic analysis (Fig. 7i). These included: (i) HM trade regulations and policies (*n* = 9); (ii) financing (*n* = 5); (iii) attributes of traditional herbalists (*n* = 4); (iv) HM quality and safety (*n* = 3); (v) HM availability and efficacy (*n* = 2); and (vi) geographical stature of the study area (*n* = 2). The national COVID-19 preventive measures (*n* = 65, 100%) were the most frequently mentioned challenge under theme (i) (Fig. 7vii). This was followed by the scarcity of some herbal medicine stocks (*n* = 58, 89.2%) which aligned with theme (v) (Fig. 7ii). The least cited challenge was the report of adverse reactions among some HM consumers (*n* = 3, 4.6%), which aligned with theme (iv) (Fig. 7v).

## Discussion

### Socio-demographic profiles of participants

The predominance of men in HM trade was also reported previously in some parts of South Africa [38], Tanzania [39], and Malawi [40]. However, in the KwaZulu-Natal, Gauteng, and Mpumalanga provinces in South Africa, the majority of the commercial herbalists were women [41]. In the current study, 36% of the participants were aged between 18 and 24 years. The age bracket of 15 to 24 years is classified as the youthful group according to the World Health Organization [42, 43]. Therefore, HM trade in Kampala city has the potential for future expansion since a large proportion of the herbalists belonged to the very active age group. The relatively high profits obtained from HM sales, as reported in this study, highlight the potential contribution of the herbal medicine industry to Uganda’s national economy, and the role these plant species play towards the attainment of the participants’ livelihood needs, primary health care services, and cultural heritage. Cunningham [44] also reported that medicinal plants constitute an important feature of the cultural, economic, medicinal, and ecological components of all cities in the world.

### Herbal medicine diversity, methods of preparation, and administration

The majority of the medicinal plant species identified in this study were in the family Fabaceae, Myricaceae and Asteraceae. Previous studies showed similar trends where Asteraceae, Fabaceae, and Cucurbitaceae were the most commonly traded botanical families in Botswana [45], and South Africa [46]. Plant families such as Fabaceae, Asteraceae, and Euphorbiaceae have the greatest number of species traded as herbal medicine possibly because these families are large and characterized by numerous species (http://www.theplantlist.org/). Plant species belonging to these families have been reported to possess numerous bioactive compounds of medical importance, such as alkaloids, quinones, saponins, anthocyanins, coumarins, flavonoids, tannins, terpenoids phenols, steroids, and stereoisomeric neolignanes [47]. Some of the plants documented in the current study, such as *C. flexuosus*, *C. limon*, and *A. sativum* have been reported to have nutritional and commercial values, and they are used in the treatment other ailments besides diarrhea and/or cough [48–51].

The number of medicinal plant species reported in the current study is generally small compared to previous ethnobotanical and/or ethnopharmacological surveys conducted in Uganda [48, 50–55]. The earlier studies were mostly based in rural settings, and focused on documenting HM used for treating all the health complications prevalent in the study communities; this resulted in higher numbers of species reported by those studies, as compared to the current study. The low number of plant species reported by the current study may be attributed to the fact that herbalists that use HM as a cardinal source of income were potentially hesitant to divulge out all the ethnopharmacological information for fear that the researchers could use this knowledge to start a similar HM business. Nevertheless, to the best of our knowledge, some of the plant species cited in the current study, such as those under family Rubiaceae, are mentioned for the first time in the management of cough and/or diarrheal diseases in this part of Uganda. These plants, for example *Gardenia ternifolia* were previously reported in for the management of other ailments though; such as the opportunistic diseases in people living with HIV/AIDs [56]. In *Gardenia ternifolia*, some bioactive compounds with inhibitory activity against deadly viruses such as the Human immunodeficiency virus (HIV), Herpes simplex virus type 1 (HSV-1), and the African swine fever virus (ASFV) were reported in earlier studies [47, 57]. Such compounds include alkaloids and flavonoids among others [47]. Studies that aim at profiling the plant species used for treating selected ailments, such as diarrhea and/or cough, can be more suitable for informing the discovery of specialized medicine for the ailments of interest, compared to those that document all the medicinal plants in a study community.

### Highly traded plant species and their parts for diarrhea and cough management

The fact that *C. limon* was cited by all the participants for the treatment of cough highlights its great potential in the management of respiratory infections. Nonetheless, its relative medical importance was not significantly different from the rest of the plant species except those with RFC less than 0.95 such as *A. indica*. The frequent use of *C. limon*, *M. foetida*, and *C. citrinus* for the management of cough as reported in the current study corroborates with previous studies in other parts of Uganda [49, 51]. Additionally, *C. flexuosus* used as a diarrhea remedy, is also an aromatic herb in hot drinks and beverages in Nepal [58]. In the current study, leaves were majorly used as medicine, followed by the stem bark. The use of leaves is commendable since this promotes sustainable utilization of the plant species and preservation of their genetic stocks, as opposed to the usage of roots, stems and/or whole plants which would rather cause obliteration of the plants [59]. However, indiscriminate plucking of leaves of highly used plant species may eventually become unsustainable [60], while use of bark may result in death of some medicinal species.

### Sources of herbal medicines traded

Forests were the main source of HM, followed by bushes and homestead farms, with minimal reports of obtaining HM stocks from wetlands and herbal shops. In Uganda, earlier studies also reported the harvesting of HM from forests [53, 61]. In the current study, the report of high dependence on Mabira Central Forest Reserve as a source of HM might be attributed to several factors: (i) it is the largest natural forest in central Uganda; (ii) it is endowed with enormous medicinal plant species diversity; (iii) relatively close proximity (54 km) to Kampala; (iv) easily accessible, and (v) it is legally acceptable to harvest non-timber vegetation resources from this forest [53, 61, 62]. The minimal dependence on herbal shops within Kampala, as sources of HM stocks, could partly be explained by the scarcity of whole sale herbal shops, which lures most herbalists to obtain medicinal plant materials directly from their natural habitats. In addition, the knowledge of HM is generally personalized and confidential [46], which potentially lures each herbalist into searching and harvesting the plants from the natural habitats.

The HM stocks were sourced from 20 (14.8%) of the 135 districts of Uganda [30], and these were evenly distributed all over the country. Hence, the findings reported in this study somewhat represent the ethnopharmacological information of the plants used for diarrhea and cough treatment in the five regions of Uganda (Central, Eastern, Northern, Western and Southern).

### Demand and supply of traded medicinal plants used for diarrhea and cough treatment in Kampala

More than half of the respondents indicated that some species were on high demand but either rare or their populations declining in the habits where they were harvested. Though the top five wild species frequently cited in the current study were not Red-listed as nationally threatened species [63], several other plants identified in this study are on the Red List [63]. These include; *C. articulata* (African Cherry Orange), *P. africana* (African Almond), and *L. trichilioides* (African Walnut), all cited in the treatment of diarrhea; as well as *M. excelsa* (African Teak), *T. indica*, and *W. ugandensis*, for cough [63]. The rising demand for many wild medicinal species leads to a potential increase in the harvesting pressure, making the affected species susceptible to local extinction. The local extinction of medicinal plant species may have global implications for human health [44]. Additionally, the recent increase in market demand for cultivated species such as *C. limon*, and *C. sinensis* is global and has been attributed to their perceived roles in the management of patients of Coronavirus disease-2019 (COVID-19) [64, 65]. Citrus fruits are some of world’s most important vegetal reservoirs of zinc, selenium, and vitamins C & D [66–69]. These minerals have been reported as effective boosters of natural immunity, and are essential in the management of respiratory viral pathogens such as SARS-Cov-2, the causative agent of COVID-19 [64, 70, 71].

Consequently, medicinal plants are known for their historical roles in counteracting the previous pandemics [72]. As such, some countries like China [73], and Uganda [74, 75] have already approved the use of herbal products as part of the medical interventions against the COVID-19 pandemic. Similarly in Madagascar, combination of A*rtemisia annua* L., *C. sinensis*, *A. sativum* and *Z. officinale* has been adopted as an important anti-COVID agent [76]. In the current study, though *A. sativum* was perceived to be abundant, it was the most expensive HM, sold at a price of UGX 48,000 ($13.15) per kilogram. This may not only be attributed to its role as a spice, but also as medicine for a wide spectrum of common ailments like respiratory diseases, gastrointestinal upsets, and cardiac complications [77, 78]. Many farmers in Uganda do not locally grow *A. sativum* but it is mostly imported from China and India [79]. The least priced medicinal plant species were sold at UGX 4000 ($1.10) per kilogram. The findings of this survey revealed a price discrepancy with the amounts previously reported in HM markets in Eastern Cape province of South Africa; on the latter, the most expensive herbal drugs were sold at $10.30 and the cheapest at $1.90 [80]. Therefore, HM trade in Kampala may offer better financial gains than in some cities elsewhere [41, 46].

### Types of herbal-selling establishments and packaging of HM in Kampala

The HM were sold in herbal shops, market stalls, pharmacies, roadside stalls, and mobile establishments. Similarly, these types of HM ventures were reported in other urban settings in south Africa [46], Kenya [81], Tanzania [82], Malaysia [83], and China [46, 84]. In Uganda, the sale of indigenous herbal products in pharmacies is symbolic of the recent widespread innovations related to improved packaging and branding of HM, comparable to the standards that are acceptable in pharmacies. Ultimately, this might raise the confidence levels among pharmacists and physicians on the use of HM in Uganda, boosting the country’s HM industry. The presence of mobile and semi-mobile HM sellers in Kampala could pose herbal safety concerns since the effective monitoring and regulation of such arrangements can be difficult [85]. Further, the re-use of packaging materials that had been discarded as wastes in Kampala, has been associated with the introduction of pathogenic microbial contaminants in HM, threatening public health elsewhere [18, 81, 86–89].

### Challenges hindering herbal medicine trade in Kampala city

The current study revealed that herbalists in Kampala operated under numerous challenges. Most of these hinderances were linked to HM regulation and policy (Fig. 7), despite the existence of legal frameworks that are supposed to streamline HM practices in the country. Such regulatory frameworks include the herbal medicine guidelines established by Uganda National Drug Authority [35], as well as the “Traditional and Complementary Medicine Act, 2019” ascended to by the president of Uganda on 14th September 2020 [90]. Other major constraints included the inadequacy of financial capital, and COVID-19 related disruptions. The disruption of economic activities by COVID-19 has also been reported globally [64, 91, 92]. Some herbalists were able to follow up their clients, hence the reports of side effects of some HM. This action is commendable since it promotes herbalist–patient/client trust and pharmacovigilance, though it is rare among herbalists worldwide [93].

## Conclusions

There is a rich diversity of medicinal plant species traded in Kampala to treat diarrhea and cough. The HM trade significantly contributes to the livelihoods of the traders in Kampala, as well as the different actors along the HM value chain throughout the country. These medicines are collected from numerous habitats especially in the wild across the country. Most of the frequently used species for management of these diseases were reported to be rare or their availability declining in their natural habitats. Therefore, in addition to the validation of the therapeutic claims, the conservation and preservation of these species is warranted. Although the trade of herbal remedies in Kampala is limited by various hindrances, most of which are linked to the policies and regulation of the herbal medicine industry, it offers a unique opportunity for rural traditional herbalists to enter the urban cash economy. Further research focusing on streamlining of herbal medicine trade, more so in urban settings, should be conducted, to support the formulation of regulatory frameworks, and to bridge the knowledge gaps in herbal medicine safety, quality, and dosages.

## Data Availability

All data generated and analyzed during this study are included in the article.

## Abbreviations

HM: Herbal medicine
TMP: Traditional medical practitioners
WHO: World Health Organization
COVID-19: Coronavirus disease-2019
KCCA: Kampala capital City Authority
